# Predictors of post-COVID-19 and the impact of persistent symptoms in non-hospitalized patients 12 months after COVID-19, with a focus on work ability

**DOI:** 10.48101/ujms.v127.8794

**Published:** 2022-08-09

**Authors:** Marta A. Kisiel, Helena Janols, Tobias Nordqvist, Jonas Bergquist, Simone Hagfeldt, Andrei Malinovschi, Magnus Svartengren

**Affiliations:** a Department of Medical Sciences, Occupational and Environmental Medicine, Uppsala University, Uppsala, Sweden; b Department of Medical Sciences, Infectious Diseases, Uppsala University, Uppsala, Sweden; c Analytical Chemistry and Neurochemistry, Department of Chemistry – BMC, Uppsala University, Uppsala, Sweden; d The ME/CFS Collaborative Research Centre at Uppsala University, Sweden; e Department of Medical Sciences, Clinical Physiology, Uppsala University, Uppsala, Sweden

**Keywords:** Post-COVID-19, long-term symptoms after COVID-19, work ability after COVID-19, healthcare workers

## Abstract

**Background:**

Better knowledge of long-term symptoms following coronavirus disease 2019 (COVID-19), the so-called post-COVID-19, in non-hospitalized patients is needed. The aim of this study was to study persisent symptoms up to 12 months after COVID-19 in non-hospitalized patients and their impact on work ability. We also investigated predictors of persistent symptoms.

**Methods:**

This study encompassed non-hospitalized adult subjects with a COVID-19 infection confirmed via positive nasopharyngeal swab polymerase chain reaction test during the first wave of the pandemic in Uppsala, Sweden. In total, 566 subjects were sent a survey via e-mail or post with an invitation to participate in the survey 12 months post-diagnosis. The majority of subjects were healthcare workers, as this group was prioritized for testing.

**Results:**

A total of 366 subjects responded, with 47% reporting persistent symptoms 12 months after their COVID-19 diagnosis. The most commonly reported symptoms at this time were impaired sense of smell and/or taste and fatigue. Among the predictors of persistent symptoms were being born abroad, lower physical fitness compared with peers before COVID-19, body mass index >25 kg/m^2^, cooccurrence of hypertension and chronic pain, and having more than seven of the general COVID-19 symptoms at the onset. Respondents with symptoms after 12 months self-reported negatively about their general health and work ability.

**Conclusions:**

This study indicated that many people who had mild COVID-19 might have a variety of long-term symptoms. It highlights the importance of considering work ability after mild COVID-19.

## Background

Researching persistent symptoms after infection with severe acute respiratory syndrome coronavirus 2 (SARS-CoV-2) has become a priority worldwide since the initial outbreak of coronavirus disease 2019 (COVID-19) (1). However, at the time of writing, there is no internationally accepted definition for this long-term condition, commonly referred to as post-COVID-19. Instead, long COVID-19 is pragmatically defined as one or a set of symptoms that can fluctuate or persist for several weeks or months after the resolution of the acute infection (2). Investigations into post-COVID-19 have been performed in distinct populations and study settings, using various measurement tools and timepoints. This makes it difficult to make direct comparative analyses or synthesize the available evidence on post-COVID-19 (3) and use the available datasets to accurately estimate the full impact of post-COVID-19 at a national, regional, or even local level.

Several recent studies have indicated that the signs of post-COVID-19 vary and included systemic, cardiovascular, pulmonary, neuropsychiatric, and nose and throat symptoms (4). It is argued that any long-term recovery challenges can be considered to be potential signs of post-COVID-19 (5). It is commonly understood that long-term symptoms can occur regardless of acute infection severity. However, acute phase severity, hospitalization, greater age, female sex, high body mass index (BMI), and any chronic somatic disease are factors associated with post-COVID-19 (6).

Early studies have indicated that post-COVID-19 can be occur even after mild acute infection (1). Our current understanding is hampered by the fact that most existing studies of acute COVID-19 have focused on hospitalized patients (7). However, it is believed that most COVID-19 patients present with mild disease that does not require hospital admission and, therefore, usually do not have any planned medical follow-up (8). Information on the long-term consequences of COVID-19 in those patients is limited (7). A few reports that included long-term follow-up in non-admitted patients suggest that 31–53% still have one or several persistent signs a year after infection (9, 10), which would translate to a significant number of people worldwide.

It could also be posited that post-COVID-19 has had and continues to have an impact on general health and may reduce the work ability of those affected. This could have an effect on the uptake or need for additional sick leave (11, 12), which could lead to further societal impact. In our previous study, we showed that there was an increased risk of staff absence among healthcare personnel and residential care workers in Sweden due to prolonged sick leave (13). However, we were not able to ascertain how post-COVID-19 affected other occupational groups.

The objective of this cross-sectional study was to evaluate persistent symptoms up to 12 months after confirmed COVID-19. We assessed whether those symptoms affected the general health and work ability of healthcare workers compared with other occupational groups based on responses to a survey sent to a group tested at the Uppsala University Hospital soon after the onset of the pandemic. Furthermore, we aimed to identify the independent predictors of persistent symptoms in non-hospitalized patients. As this study was based on the general population tested in the first wave of the pandemic in Sweden, when healthcare staff were prioritized for SARS-CoV-2 testing, even in case of mild symptoms, most subjects in the study population were healthcare workers.

## Methods

### Study design and study population

This one-year follow-up cohort study is part of a prospective longitudinal cohort study, COMBAT post-COVID-19, conducted at the Uppsala University Hospital. A questionnaire was sent to all adult non-hospitalized COVID-19 patients (≥18 years old) diagnosed at the Department of Infectious Diseases between March 10 and July 30, 2020. Other eligibility criteria included a confirmed diagnosis based on a positive nasopharyngeal swab polymerase chain reaction (PCR) test for SARS-CoV-2. All tested patients were symptomatic.

**Figure 1:**
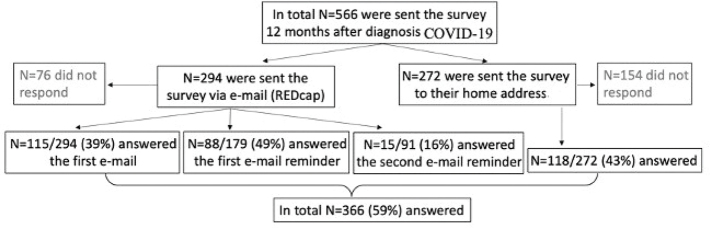
Flowchart of participants. In total, 566 non-hospitalized COVID-19 patients were sent the questionnaire (e-mail via REDcap or to home address) 12 months after their COVID-19 diagnosis. A total of 366 patients responded and made up the study population.

The patients were identified via the electronic medical records database. Identified candidates were sent the questionnaire 51–54 weeks after their positive test result. The surveys were primarily sent by e-mail via the widely used REDcap application, which is considered a secure data capturing tool for clinical research (14). If no e-mail address was available for a patient, a survey and a return envelope were sent to the patient’s home address through the regular postal service. The e-mail survey respondents received up to two reminders, one and two weeks after the initial contact was made (Figure 1). Those receiving postal surveys did not get any reminder, as it would not reach them during the relevant timeframe.

All respondents gave a digital or written informed consent at the start of the questionnaire. This study was approved by the Institutional Ethics Committee of the Uppsala University Hospital (EPN number: 2020-05707) and conducted in accordance with the Helsinki Declaration.

### Definition of post-COVID-19

In this study, post-COVID-19 was defined as symptoms persisting at least 12 weeks (3 months) after disease onset, in accordance with the National Institute for Health and Care Excellence (NICE) criteria (15).

### Included data

Captured data included age (stratified into three age groups: 18–35, 36–55, and >55 years) and sex, as encoded in each patient’s personal identity number.

The survey included questions on the following (full survey shown in the supplementary materials):

- Marital status.
- Country of birth and countries of parents’ birth.
- Education level classified into three categories: elementary school/upper secondary school, vocational college (at least two years education not equivalent to an academic degree), and university qualification of at least 3 years.
- Smoking status, categorized as current smokers, former smokers, and non-smokers.
- Use of snuff.
- Height and weight, used to calculate BMI, which was classified as underweight <18.5 kg/m^2^, normal weight 18.5–24.99 kg/m^2^, overweight 25–29.99 kg/m^2^, obese ≥30 kg/m^2^.
- Physician-diagnosed common chronic diseases such as hypertension, other heart disease, hypo-/hyperthyroidism, diabetes mellitus, lung disease, liver disease, immunosuppressive treatment, cancer, stroke, depression, anxiety, and chronic pain.
- Self-reported physical fitness before COVID-19 and 12 months after COVID-19 in comparison to persons of the same age (classified as worse, same, or better).
- Symptoms at the COVID-19 onset.
- Symptoms 1, 3, 6, and 12 months after COVID-19. Symptoms at the onset and afterward were assessed using a checklist of 20 self-reported symptoms, adopted from the WHO/ISARIC platform (16).
- Having sought health care due to long-term symptoms following COVID-19.
- Self-reported general health before and 12 months after COVID-19.
- Self-reported work ability before and 12 months after COVID-19.
- Work status in primary occupation: work/studies/parental leave/sick leave/unemployed/retired.
- The occupation further categorized into health care with patient contact (including physicians, nurses, assistant nurses, occupational therapists, physiotherapists, and psychologists) and other occupations (including health care without patient contact, e.g. administrators working in hospital in primary care; other white-collar workers; blue-collar workers performing manual labor or in occupations requiring only an elementary education). The classification of workers was based on a Swedish standard system for classification of occupations (Standard för Svensk Yrkesklassificering 2012, SSYK2012) (17).
- Self-reported sick leave during the year before the pandemic and the duration of any such sick leave.
- Self-reported sick leave due to COVID-19 and its duration.

### Statistical analysis

Categorical variables are presented as proportions, and continuous variables are presented as means with standard deviation (SD). The mean differences of work ability and general health before and 12 months after COVID-19 were assessed with 95% confidence intervals (CIs) (normally approximated as 1.96×standard error) in two occupational groups: healthcare workers with patient contact and others. Mean score differences between occupational groups were compared with t-tests. The predictors of symptoms after 12 months were calculated with relative risk (RR) and 95% CIs. The chi-squared test or Fisher’s test was used to calculate *P* values. A *P* value <0.05 was considered significant. The analyses were performed in Excel and SAS 9.4.

## Findings

### Basic characteristics

A total of 566 patients (72% female) met the inclusion criteria, with 336 (59%) answering the survey. The study participants were between 19 and 87 years (mean age 43.1 years, SD 13.4). Females represented 75.3% (*n* = 253) of the study participants. The response frequency is shown in the flowchart (Figure 1). There were 158 patients (47%) who reported persistent symptoms 12 months after their COVID-19 diagnosis (Figure 1). There was a slightly higher proportion of females in the group with persistent symptoms (79%) compared with those without symptoms (72%). In the total respondent cohort, 305 subjects (91%) were in employment when they answered the survey, with the majority (*n* = 234, of whom *n* = 174 were female) working in health care with patient contact. The other occupational groups included 34 workers (*n* = 26 female) in health care without patient contact (administrators), 25 (*n* = 15 female) other white-collar workers, 18 (*n* = 9 female) blue-collar workers, and four subjects who did not report their occupation (Table 1). Persistent symptoms at 12 months were reported by 82 females and 17 males working in health care with patient contact (53% of females vs. 34% of males in this field). However, in the other occupational groups, there were 25 females and 14 males who had post-COVID-19 (50% of the females and 49% of the males in the other occupational groups).

**Table 1:** Characteristics of the study population grouped based on the presence or absence of symptoms at 12 months post-infection. Data presented as *n* and percent (%) for categorical variables and as mean and standard deviation (SD) for continued variables.

Data used	Missing answer(s)	No symptoms *n* = 178 (53%)	Symptoms *n* = 158 (47%)
Sex	0		
Female		128 (72)	125 (79)
Male		50 (28)	33 (21)
Age group (years)	0		
18–35		58 (33)	42 (27)
36–55		69 (39)	74 (47)
>55		51 (28)	42 (26)
Marital status	1		
Married		48 (27)	50 (32)
Living with partner		78 (44)	53 (33)
Divorced		30 (17)	38 (24)
Widow/-er		9 (5)	5 (3)
Single		12 (7)	12 (8)
Country of birth	5		
Born in Sweden with two Swedish parents		134 (75)	104 (66)
Born in Sweden with one Swedish parent		14 (8)	11 (7)
Born in Sweden with no Swedish parents		5 (3)	6 (4)
Not born in Sweden		23 (13)	34 (22)
Education	1		
Elementary school/upper secondary school		53 (29.8)	60 (37.9)
Vocational education		15 (8.4)	14 (8.9)
University		110 (61.8)	83 (52.5)
Work/other activity	8		
Current work		164	141
Healthcare with patient contact*		125	99
Healthcare without patient contact		19	15
Other white-collar		11	14
Blue-collar		8	10
Occupation not reported		1	3
No current work/other			
Sick leave		3 (2)	3 (2)
Parental leave		4 (2)	1 (1)
Unemployed		0	1 (1)
Retired		4 (2)	4 (2)
Student		2 (1)	1 (1)
Smoking status	3		
Current		6 (3)	6 (4)
Former		32 (18)	34 (21)
Never		137 (77)	118 (75)
Snuff	5	33 (19)	32 (20)
BMI (kg/m^2^)	18		
Underweight < 18.5		1 (1)	1 (1)
Normal 18.5–24.99		104 (58)	70 (44)
Overweight 25–29.99		43 (24)	50 (32)
Obesity > 30		20 (11)	25 (16)
Comorbidity	0		
Hypertension		15 (8)	21 (13)
Other heart disease		3 (2)	5 (3)
Hypo-/hyperthyroidism		10 (6)	14 (9)
Diabetes mellitus		5 (3)	6 (4)
Lung disease		14 (8)	20 (13)
Immunosuppressive treatment		5 (3)	10 (6)
Cancer		7 (4)	10 (6)
Stroke		1 (1)	2 (1)
Depression		25 (14)	24 (15)
Anxiety		26 (15)	23 (15)
Chronic pain		3 (2)	10 (6)
Number of symptoms at onset	4		
1–2		34 (19)	13 (8)
3–4		48 (27)	28 (18)
5–6		48 (27)	31 (20)
7 or more		46 (26)	84 (53)
Physical fitness compared with same-age peers before COVID-19	4		
Worse		20 (11)	42 (27)
Same		107 (60)	74 (47)
Better		48 (27)	41 (26)
Physical fitness compared with same-age peers after COVID-19	3		
Worse		28 (16)	81 (51)
Same		107 (60)	58 (37)
Better		42 (24)	17 (11)
Sought healthcare due to persistent symptoms during the follow-up period		17 (9)	50 (32)
Self-reported sick leave during the year prior to the pandemic		44 (25)	37 (23)
Number of weeks, mean (SD)		2.3 (3)	3.7 (6)
Self-reported sick leave due to COVID-19 during the follow-up period		27 (15)	55 (35)
Number of weeks, mean (SD)		4 (4.6)	8.1(6.5)

* Currently working in healthcare with patient contact (includes physicians, nurses, assistant nurses, occupational therapists, physiotherapists, and psychologists).

### Distribution of symptoms after 12 months

**Figure 2:**
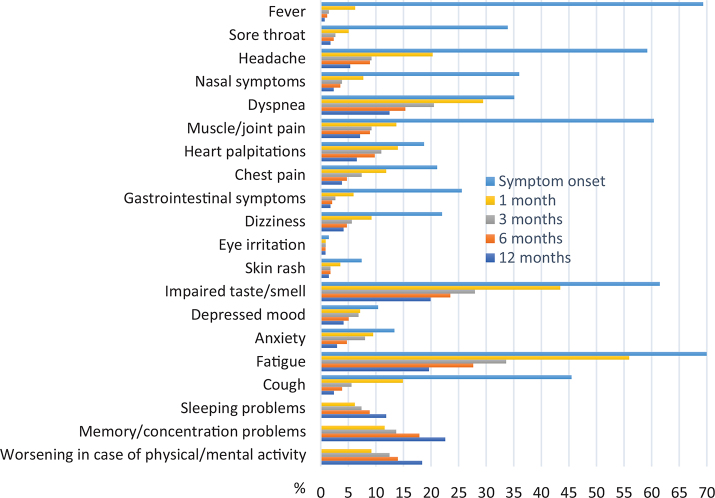
Distribution of symptoms (%) over time, from symptom onset through 1, 3, 6, and 12 months after COVID-19 diagnosis in the whole study population.

Figure 2 shows the distribution of symptoms over time, from the initial onset through 1, 3, 6, and 12 months after COVID-19. Acute symptoms, such as fever, sore throat, and coughing, tended to improve faster than chronic symptoms, like fatigue, depression, anxiety, and problems with memory or concentration. Memory and concentration problems, sleeping problems, and worsening of symptoms due to mental or physical activity increased over time in the respondent cohort. The most common symptoms at 12 months post-infection were impaired sense of taste and/or smell and fatigue, followed by memory and/or concentration problems and dyspnea. Less common symptoms were skin rash, eye irritation, and gastrointestinal symptoms. The distributions of all symptoms, except depression and anxiety, were comparable over time among healthcare workers with patient contact and other occupational groups (Supplementary Fig. 1). Healthcare workers reported a faster reduction rate of depression and anxiety at the follow-up than the other occupational groups. However, a low proportion of subjects in both occupational groups reported such symptoms at 12 months. The most common comorbidities in both occupational groups were depression, anxiety, and hypertension (Supplementary Table 1).

### Consequences of persistent symptoms

Among respondents with persistent symptoms (51%) 12 months after contracting COVID-19, significantly more experienced lower physical fitness levels at this timepoint compared with peers without symptoms (51% vs. 16%) (*P* < 0.01). Also, a significantly higher proportion of subjects with persistent symptoms (35%) reported taking sick leave due to COVID-19 during the first year compared with those without symptoms (15%) (*P* < 0.001).

**Figure 3:**
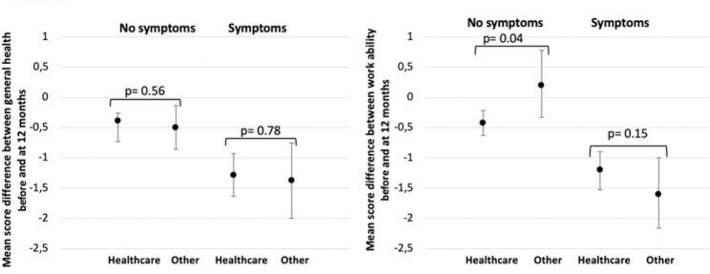
Mean score difference with 95% confidence intervals of self-reported health and work ability before COVID-19 and 12 months after infection among working respondents (*n* = 305) with or without symptoms 12 months after the infection, divided into two groups: healthcare with patient contacts (health care) and other occupational groups (other). A difference score under ‘0’ means that subjects reported lower general health or work ability at 12 months, and this was equal to the negative impact of the COVID-19 infection. The *P* values were calculated with t-tests and a *P* value < 0.05 was considered significant.

The two occupational categories had comparable RRs of persistent symptoms after 12 months (Figure 3). In addition, symptoms persisting at 12 months affected the general health and work ability of all subjects (Figure 3). This was slightly less pronounced for healthcare workers with patient contact than those in other occupations. Work ability in respondents with no persistent symptoms at the 12-month follow-up had decreased among healthcare workers and increased in other occupations, causing a significant gap in the work ability between those employed in health care and other occupational groups.

### Predictors of persistent symptoms

Table 2 shows independent variables as predictors of persistent symptoms 12 months after a COVID-19 diagnosis. It appeared that those born outside Sweden, with both parents also born outside Sweden, had a significantly increased RR of long-term symptoms. Also, subjects with lower education, such as only elementary school and upper secondary school, tended to report persistent symptoms 12 months after the infection. Bodyweight (BMI > 25 kg/m^2^) was a significant predictor of long-term symptoms. In terms of comorbidities, only self-reported and pre-diagnosed hypertension and chronic pain were indicators of a significant increase in RR for persistent symptoms. In addition, seven or more symptoms at disease onset increased the RR for the patient to have persistent symptoms during the 12-month period after contracting COVID-19. Those who self-reported poor physical fitness before COVID-19 seemed to have a higher risk of having long-term symptoms. However, sick leave during the year before the pandemic was not linked to an increased risk of persistent symptoms 12 months after COVID-19.

**Table 2:** Predictors of symptoms after 12 months were calculated as relative risk (RR) and 95% confidence intervals (95% CIs). The chi-squared test or Fisher’s test was used to calculate P values. A P value < 0.05 was considered significant.

Predictors	RR (95% CI)	*P* value
Sex/female	1.24 (0.93–1.66)	0.12
Age group (years)		
18–35	1	Reference
36–55	1.23 (0.93–1.63)	0.13
>55	1.07 (0.78–1.50)	0.65
Marital status		
Married	1	Reference
Partner	0.79 (0.60–1.51)	0.12
Divorced	1.09 (0.82–1.46)	0.53
Widow/-er	0.70 (0.34–1.45)	0.13
Single	0.98 (0.62–1.52)	0.93
Country of birth		
Both parents and respondent born in Sweden	1	Reference
One parent and respondent born in Sweden	1.01 (0.63–1.60)	0.97
None of parents but respondent born in Sweden	1.25 (0.71–1.18)	0.48
Respondent not born in Sweden	1.36 (1.05–1.77)	0.03
Education		
Elementary school/upper secondary school	1.23 (0.97–1.56)	0.06
Vocational college	1.12 (0.74–1.70)	0.59
University	1	Reference
Occupation		
Healthcare with patient contact	1	Reference
Other occupation	0.91 (0.71–1.18)	0.50
Smoking status		
Current	1.08 (0.60-1.93)	0.80
Former	1.11 (0.85–1.46)	0.45
Never	1	Reference
Snuff	1.05 (0.80–1.40)	0.70
BMI (kg/m^2^)		
Underweight < 18.5	1.24 (0.31–5.03)	0.48
Normal 18.5–24.99	1	Reference
Overweight 25–29.99	1.33 (1.03–1.74)	0.03
Obesity ≥ 30	1.38 (1.00–1.90)	0.06
Comorbidity		
Hypertension	1.34 (1.00–1.78)	0.06
Other heart disease	1.34 (0.78–2.32)	0.20
Hypo-/hyperthyroidism	1.26 (0.89–1.80)	0.08
Diabetes mellitus	1.17 (0.67–2.02)	0.19
Lung disease	1.29 (0.95–1.75)	0.25
Immunosuppressive treatment	1.45 (0.99–2.10)	0.21
Cancer	1.27 (0.84–1.92)	0.14
Depression/anxiety	1.05 (0.80–1.36)	0.34
Chronic pain	1.68 (1.21–2.31)	0.03
Number of symptoms at onset		
1–2	1	Reference
3–4	1.33 (0.77–2.30)	0.29
5–6	1.42 (0.83–2.42)	0.18
7 or more	2.33 (1.44–3.78)	< 0.01
Physical fitness compared with same-age peers before COVID-19		
Worse	1.47 (1.11–1.95)	< 0.01
Same	0.89 (0.67–1.18)	0.42
Better	1	Reference
Self-reported sick leave during the year prior to the pandemic (reference group was subjects without symptoms 12 months after COVID-19)	0.96 (0.73–1.27)	0.81

## Interpretation

This study found that 47% of the non-hospitalized patient respondents reported persistent symptoms 12 months after contracting COVID-19. The most commonly reported symptoms at this timepoint were fatigue and impaired sense of smell and/or taste, memory and concentration problems, and shortness of breath. While the prevalence rates of most symptoms decreased over time, the rates of fatigue, memory and concentration problems, and sleeping problems increased over time. Furthermore, we showed that factors such as non-Swedish ethnic origin, having seven or more symptoms at disease onset, and several other medical or general health issues, such as low physical fitness, BMI > 25 kg/m^2^, and suffering from hypertension and chronic pain, were significant predictors of persistent symptoms at 12 months after COVID-19. Also, more subjects with low education, such as only elementary school and upper secondary school, appeared to experience persistent symptoms.

Almost half of the respondents in this population had at least one persistent symptom 12 months after infection. This finding was consistent with results from a telephone-based Italian survey study that looked only at non-admitted patients 12 months post-infection (15). This study found that 53% of 304 patients who were treated at home had persistent symptoms 12 months after COVID-19. Conversely, a report based on the same questionnaire used in this study, but which also gathered data on severity of COVID-19 infection, showed that 31% of 99 patients had persistent symptoms 12 months after non-hospitalized infection, whereas 64% of 145 with a moderate infection and 87% of 98 severe/critical hospitalized survivors had persistent symptoms (14). In line with our study, a Danish population study of non-hospitalized COVID-19 patients reported that the most common persistent symptoms at 4 and 12 weeks after infection were fatigue and impaired sense of smell and/or taste (18). These symptoms were also those most frequently reported in our patients at 12 months.

Other findings revealed that the distribution of persistent symptoms varied over time. Acute symptoms like fever declined, while fatigue and memory issues increased over time. Davis et al. reported similar findings in a large population study that concentrated mainly on non-hospitalized COVID-19 patients (11). In line with the meta-analysis, our results indicated that the most common symptoms at the onset of a COVID-19 infection in non-hospitalized patients were fever, fatigue, ageusia, insomnia, headache, and muscle and joint pains (19).

Several studies have shown that females are more often affected by post-COVID-19 than males (20, 21). In our study, there was a slightly higher proportion of females (79%) in the group experiencing symptoms after 12 months compared with the group with no symptoms (72%). However, sex did not increase the RR of experiencing persistent symptoms at 12 months. This may at least partially be explained by the higher response rate from female patients (75%). In line with the previous study (18), we showed that there was a higher proportion of females than males with persistent symptoms in the group of healthcare workers with patient contact. We also found that a comparable percentage of females and males working in other occupations experienced persistent symptoms at 12 months.

Our study indicated that more respondents who were not born in Sweden and whose parents also came from another country had persistent symptoms 12 months post-infection. Also, the subjects with the lowest education levels tended to have persistent symptoms after 12 months. This finding seems to be in agreement with those of similar studies, where lower education and having a non-Swedish ethnic background were associated with a higher risk of post-COVID-19 (21). In addition, as shown by Karmakar et al. and Drefahl et al. using population-based cohorts in the United States and Sweden, respectively, there was a strong correlation between being part of a minority community and being vulnerable to COVID-19 infection, hospitalization, and mortality (22, 23). Suggested explanations include that the immigrant population might not understand all the information from the authorities regarding the pandemic and might also have less trust in healthcare services (21).

The risk of persistent symptoms was higher among the overweight (BMI > 25 kg/m^2^) and obese (BMI > 30 kg/m^2^) than among those of normal weight. These findings were in line with those of previous long-term sequelae studies among patients after mild COVID-19 (10) as well as among those hospitalized with COVID-19 (24). Augustin et al. found that patients presenting with post-COVID-19 symptoms after hospitalization had a higher BMI compared with those not hospitalized (25). Overweight and obesity have been found to correlate with higher susceptibility to contracting COVID-19 leading to hospital admissions, length of hospital stay, and risk of mortality (26). Fernandez-de-Las-Penas et al. found in their study that a BMI > 30 kg/m^2^ could be independently linked to a larger number of post-COVID-19 symptoms, including poor sleep quality, 7 months after hospitalization due to COVID-19 (27).

As regards general fitness levels, self-reporting lower physical fitness compared with peers before contracting COVID-19 was a predictor of symptoms 12 months after infection. This was in line with the results of a previous study, which demonstrated that low levels of physical activity before contracting COVID-19 increased the risk of severe acute infection, hospital admission, and even mortality (28). The UK Biobank study found that those who reported higher levels of physical activity were better protected against poor COVID-19 outcomes independently of age, sex, BMI, and smoking (29). This was also seen from general health or population-level health surveys performed before the pandemic, which showed that moderate physical activity reduced the risk of respiratory virus infections and speeded recovery compared with low physical activity (30).

We found that patients with chronic comorbidities like hypertension and lung disease had increased risk of post-COVID-19 in the 12 months after the infection. These are common somatic comorbidities that have been found to increase the risk of post-COVID-19 in previous studies of long-term sequelae in non-admitted patients (18). However, we did not find an association between coexisting depression and/or anxiety and persistent symptoms after 12 months. This indicates that long-term symptoms following COVID-19 are not due only to somatic disease or social or psychological distress related to the pandemic and lockdowns.

Another aspect that could be compared with findings from other studies of non-admitted COVID-19 patients was the risk for persistent symptoms after 12 months among those working in health care with patient contact versus in other occupational groups (18). We found that respondents with persistent symptoms at 12 months were more affected in terms of both work ability and general health compared with those without symptoms at this timepoint. This is in line with past studies, demonstrating that even mild infection may lead to a substantial reduction in an individual’s ability to work, both in the general population (11) and among healthcare personnel (12). Surprisingly, healthcare personnel without symptoms also reported a reduced work ability. This might be explained by poor working conditions for healthcare workers during the pandemic (13).

One of the strengths of this study is that this is one of the first studies on long COVID-19 among non-hospitalized patients with a long-term follow-up (1 year). In addition, it includes a broad range of sociodemographic factors and compares responses from different occupational groups. This adds to the limited evidence from two previous studies regarding persistent symptoms in non-hospitalized patients after one year (14, 15). Our study has the advantage of having a relatively homogeneous patient group with laboratory-confirmed infection at the beginning of the pandemic, allowing a comparison between different occupational groups.

One limitation of this study was the response frequency of 59%. It could be surmised that subjects without persistent symptoms might be less eager to complete and/or return the questionnaire. There was a higher proportion of respondents in the e-mail group. However, response frequencies were similar after the original invitation. Those contacted via e-mail received two reminders, whereas reminders were not sent to the postal group. The response frequency is in line with those generally seen for epidemiological studies in Sweden (31).

On a cautionary note, it should be considered that the retrospective nature of reporting symptoms at onset and 1, 3, and 6 months after infection or asking questions regarding the time before the infection might create recall bias. However, it can be observed from studies that we can follow patients with multiple phone calls at various timepoints if only a small proportion of respondents participate (32). Other self-reported information, such as weight and comorbidity, might also create bias. In addition, we did not ask about treatment during the acute COVID-19 infection or whether patients were concurrently taking medication for any chronic diseases. Moreover, the generalizability of the results is limited, as this study was performed at a single institution. At the time of this study, the general recommendation was that persons presenting with mild disease should stay at home if they could. Occupational groups other than healthcare workers were less likely to be tested for COVID-19 during the first months of the pandemic, which may have skewed the results.

In summary, this study indicated that many people who initially presented with mild COVID-19 might live with long-term symptoms. The persistent symptoms, which can change over time, confirm that post-COVID-19 has a multi-systemic involvement even after mild COVID-19 infection in healthy younger individuals. The main novelty of this study was that we showed that persistent symptoms led to a reduction of work ability in both healthcare workers with patient contact and other occupations. This gives impetus to the idea that multidisciplinary teams assessing long COVID-19 need to include occupational health specialists to support workers suffering from long-term symptoms. As the pandemic has not yet been declared to have ended and the availability of testing for SARS-CoV-2 has improved greatly, further studies are needed to determine the long-term impact of long COVID-19 in various occupational groups.

## Funding

The Open Medicine Foundation (JB) is acknowledged for support. The Åke Wiberg stiftelse (MK), Tore Nilsons stiftelse (MK), and Stiftelsen Lars Hiertas Minne (MK) are also acknowledged for support.

## Data sharing

We can share an anonymized database after publication.

## Notes on contributors

***Marta A. Kisiel***, MD PhD, Department of Medical Sciences, Occupational and Environmental Medicine, Uppsala University, Uppsala, Sweden.

***Helena Janols***, MD PhD, Department of Medical Sciences, Infectious Diseases, Uppsala University, Uppsala, Sweden.

***Tobias Nordqvist***, PhD, Department of Medical Sciences, Occupational and Environmental Medicine, Uppsala University, Uppsala, Sweden.

***Jonas Bergquist***, Professor, Analytical Chemistry and Neurochemistry, Department of Chemistry – BMC, Uppsala University, Uppsala, Sweden.

***Simone Hagfeldt***, The ME/CFS Collaborative Research Centre at Uppsala University, Sweden.

***Andrei Malinovschi***, Professor, Department of Medical Sciences, Clinical Physiology, Uppsala University, Uppsala, Sweden.

***Magnus Svartengren***, Professor, Department of Medical Sciences, Occupational and Environmental Medicine, Uppsala University, Uppsala, Sweden.

## ORCID

Marta A. Kisiel https://orcid.org/0000-0002-0410-1509

Helena Janols https://orcid.org/0000-0002-3857-163X

Tobias Nordquist https://orcid.org/0000-0002-4864-9488

Jonas Beerquist https://orcid.org/0000-0002-6259-3804

Andrei Malinovschi https://orcid.org/0000-0002-4098-7765

Magnus Svartengren https://orcid.org/0000-0002-8165-7236

## Declaration of interests

The authors report no conflicts of interest.

## Supplementary Material

Predictors of post-COVID-19 and the impact of persistent symptoms in non-hospitalized patients 12 months after COVID-19, with a focus on work ability

Predictors of post-COVID-19 and the impact of persistent symptoms in non-hospitalized patients 12 months after COVID-19, with a focus on work ability

Predictors of post-COVID-19 and the impact of persistent symptoms in non-hospitalized patients 12 months after COVID-19, with a focus on work ability

## References

[1] Greenhalgh T, Knight M, A’Court C, Buxton M, Husain L. Management of post-acute Covid-19 in primary care. BMJ 2020;370:m3026. doi: 10.1136/bmj.m3026 32784198

[2] Dennis A, Wamil M, Alberts J, Oben J, Cuthbertson DJ, Wootton D, et al. Multiorgan impairment in low-risk individuals with post-COVID-19 syndrome: a prospective, community-based study. BMJ Open 2021;11:e048391. doi: 10.1101/2020.10.14.20212555 PMC8727683 33785495

[3] Iqbal FM, Lam K, Sounderajah V, Clarke JM, Ashrafian H, Darzi A. Characteristics and predictors of acute and chronic post-COVID syndrome: a systematic review and meta-analysis. EClinicalMedicine 2021;36:100899. doi: 10.1016/j.eclinm.2021.100899 34036253 PMC8141371

[4] Viet-thi T, Porcher R, Pane I, Ravaus P. Course of post COVID-19 disease symptoms over time in the ComPaRe long COVID prospective e-cohort. Nat Communication 2022;13:1812. doi: 10.1038/s41467-022-29513-z PMC8983754 35383197

[5] Carfi A, Bernabei R, Landi F. Gemelli against C-P-ACSG. Persistent symptoms in patients after acute COVID-19. JAMA 2020;324:603–605. doi: 10.1001/jama.2020.12603 32644129 PMC7349096

[6] Carvalho-Schneider C, Laurent E, Lemaignen A, Beaufils E, Bourbao-Tournois C, Laribi S, et al. Follow-up of adults with noncritical COVID-19 two months after symptom onset. Clin Microbiol Infect 2021;27:258–263. doi: 10.1001/jama.2020.12603 33031948 PMC7534895

[7] Fernandez-De-Las-Penas C, Palacios-Cena D, Gomez-Mayordomo V, Florencio LL, Cuadrado ML, Plaza-Manzano G, et al. Prevalence of post-COVID-19 symptoms in hospitalized and non-hospitalized COVID-19 survivors: a systematic review and meta-analysis. Eur J Intern Med 2021;92:55–70. doi: 10.1016/j.ejim.2021.06.009 34167876 PMC8206636

[8] Wu Z, McGoogan JM. Characteristics of and important lessons from the coronavirus disease 2019 (COVID-19) outbreak in China: summary of a report of 72314 cases from the Chinese Center for Disease Control and Prevention. JAMA 2020;323:1239–1242. doi: 10.1001/jama.2020.2648 32091533

[9] Wynberg E, Van Willigen HDG, Dijkstra M, Boyd A, Kootstra NA, Van Den Aardweg JG, et al. Evolution of COVID-19 symptoms during the first 12 months after illness onset. Clin Infect Dis 2021:ciab759. doi: 10.1101/2021.05.05.21256710 34473245 PMC8522402

[10] Boscolo-Rizzo P, Guida F, Polesel J, Marcuzzo AV, Capriotti V, D’Alessandro A, et al. Sequelae in adults at 12 months after mild-to-moderate coronavirus disease 2019 (COVID-19). Int Forum Allergy Rhinol 2021;11:1685–1688. doi: 10.1002/alr.22832 34109765 PMC9291310

[11] Davis HE, Assaf GS, McCorkell L, Wei H, Low RJ, Re’em Y, et al. Characterizing long COVID in an international cohort: 7 months of symptoms and their impact. EClinicalMedicin. 2021;38:101019. doi: 10.1016/j.eclinm.2021.101019 PMC8280690 34308300

[12] Havervall S, Rosell A, Phillipson M, Mangsbo SM, Nilsson P, Hober S, et al. Symptoms and functional impairment assessed 8 months after mild COVID-19 among health care workers. JAMA 2021;325:2015–2016. doi: 10.1001/jama.2021.5612 33825846 PMC8027932

[13] Kisiel MA. Nordqvist T, Westman G, Svartegren M, Malinovschi A, Janols H. Patterns and predictors of sick leave among Swedish non-hospitalized healthcare and residential care workers with Covid-19 during the early phase of the pandemic. PLoS One 2021;16:e0260652. doi: 10.1371/journal.pone.0260652 34882720 PMC8659339

[14] Lyon JA, Garcia-Milian R, Norton HF, Tennant MR. The use of research electronic data capture (REDCap) software to create a database of librarian-mediated literature searches. Med Ref Serv Q 2014;33:241–252. doi: 10.1080/02763869.2014.925379 25023012 PMC4339087

[15] The National Institute for Health and Care Excellence (NICE). COVID-19 rapid guidelines, England, 2021, accessed 2022-07-25. 33497154

[16] WHO/ISARIC. COVID-19 case record form. Global COVID-19: clinical platform. novel coronavirus (COVID-19) rapid version. 2020. Available from: https://apps.who.int/iris/handle/10665/331768, accessed 2022-07-25

[17] SSYK2012. Standard för svensk yrkesklassificering 1996. 2012. Available from: https://www.scb.se/dokumentation/klassifikationer-och-standarder/standard-for-svensk-yrkesklassificering-ssyk/, accessed 2022-07-25

[18] Bliddal S, Banasik K, Pedersen OB, Nissen J, Cantwell L, Schwinn M, et al. Acute and persistent symptoms in non-hospitalized PCR-confirmed COVID-19 patients. Sci Rep. 2021;11:13153. doi: 10.1038/s41598-021-92045-x 34162913 PMC8222239

[19] Fernandez-De-Las-Penas C, Palacios-Cena D, Gomez-Mayordomo V, Florencio LL, Cuadrado ML, Plaza-Manzano G, et al. Prevalence of post-COVID-19 symptoms in hospitalized and non-hospitalized COVID-19 survivors: a systematic review and meta-analysis. Eur J Intern Med 2021;92:55–70. doi: 10.1016/j.ejim.2021.06.009 34167876 PMC8206636

[20] Augustin M, Schommers P, Stecher M, Dewald F, Gieselmann L, Gruell H, et al. Post-COVID syndrome in non-hospitalised patients with COVID-19: a longitudinal prospective cohort study. Lancet Reg Health Eur 2021;6:100122. doi: 10.1016/j.lanepe.2021.100122 34027514 PMC8129613

[21] Einvik G, Dammen T, Ghanima W, Heir T, Stavem K. Prevalence and risk factors for post-traumatic stress in hospitalized and non-hospitalized COVID-19 patients. Int J Environ Res Public Health 2021;18:2079. doi: 10.3390/ijerph18042079 33672759 PMC7924607

[22] Karmakar M, Lantz PM, Tipirneni R. Association of social and demographic factors with COVID-19 incidence and death rates in the US. JAMA Netw Open 2021;4:e2036462. doi: 10.1001/jamanetworkopen.2020.36462 33512520 PMC7846939

[23] Drefahl S, Wallace M, Mussino E, Aradhya S, Kolk M, Branden M, et al. A population-based cohort study of socio-demographic risk factors for COVID-19 deaths in Sweden. Nat Commun 2020;11:5097. doi: 10.1038/s41467-020-18926-3 33037218 PMC7547672

[24] Aminian A, Bena J, Pantalone KM, Burguera B. Association of obesity with postacute sequelae of COVID-19. Diabetes Obes Metab. 2021;23:2183–2188. doi: 10.1111/dom.14454 34060194 PMC8239834

[25] Augustin M, Lehmann C. Post-COVID syndrome: turning convalescence into illness? – Authors’ reply. Lancet Reg Health Eur 2021;7:100170. doi: 10.1016/j.lanepe.2021.100170 34308410 PMC8275030

[26] Sjogren L, Stenberg E, Thuccani M, Martikainen J, Rylander C, Wallenius V, et al. Impact of obesity on intensive care outcomes in patients with COVID-19 in Sweden-A cohort study. PLoS One 2021;16:e0257891. doi: 10.1371/journal.pone.0257891 34644316 PMC8513867

[27] Fernandez-De-Las-Penas C, Torres-Macho J, Elvira-Martinez CM, Molina-Trigueros LJ, Sebastian-Viana T, Hernandez-Barrera V. Obesity is associated with a greater number of long-term post-COVID symptoms and poor sleep quality: a multicentre case-control study. Int J Clin Pract 2021;75:e14917. doi: 10.1111/ijcp.14917 34569684 PMC8646300

[28] Sallis R, Young DR, Tartof SY, Sallis JF, Sall J, Li Q, et al. Physical inactivity is associated with a higher risk for severe COVID-19 outcomes: a study in 48 440 adult patients. Br J Sports Med 2021;55:1099–1105. doi: 10.1136/bjsports-2021-104080 33849909

[29] Zhang X, Li X, Sun Z, He Y, Xu W, Campbell H, et al. Physical activity and COVID-19: an observational and Mendelian randomisation study J Glob Health 2020;10:020514. doi: 10.7189/jogh.10.020514 33312507 PMC7719276

[30] Matthews CE, Ockene IS, Freedson PS, Rosal MC, Merriam PA, Hebert JR. Moderate to vigorous physical activity and risk of upper-respiratory tract infection. Med Sci Sports Exerc 2002;34:1242–1248. doi: 10.1097/00005768-200208000-00003 12165677

[31] Kuiper IN, Svanes C, Benediktsdottir B, Bertelsen RJ, Braback L, Dharmage SC, et al. Agreement in reporting of asthma by parents or offspring - the RHINESSA generation study. BMC Pulm Med 2018;18:122. doi: 10.1186/s12890-018-0687-4 30053806 PMC6062946

[32] Petersen MS, Kristiansen MF, Hanusson KD, Danielsen ME, Bjarni AS, Gaini S, et al. Long COVID in the Faroe Islands - a longitudinal study among non-hospitalized patients. Clin Infect Dis 2020;73:e4058–e4063. doi: 10.1093/cid/ciaa1792 PMC7799340 33252665

