# Supplementary material for: Predictors of post-COVID-19 and the impact of persistent symptoms in non-hospitalized patients 12 months after COVID-19, with a focus on work ability
Source: Ups J Med Sci. 2022 Aug 9;127:10.48101/ujms.v127.8794. doi: 10.48101/ujms.v127.8794 (PMC9383047; doi:10.48101/ujms.v127.8794)
Supplement: Predictors of post-COVID-19 and the impact of persistent symptoms in non-hospitalized patients 12 months after COVID-19, with a focus on work ability [file UJMS-127-8794-s001.pdf]

The questions from the survey used in this study.

**Question**	**Response options**
Marital status (tick one or more options)	☐ Married ☐ Partner ☐ Divorced or separated ☐ Widow/widower
Were you born in Sweden?	☐ Yes ☐ No
Was your mother born in Sweden?	☐ Yes ☐ No
Was your father born in Sweden?	☐ Yes ☐ No
Which in your highest educational level?	☐ Elementary school<br>☐ Upper secondary school<br>☐ Vocational college at least 2 years<br>☐ University at least 3 years
Have/do you smoked cigarettes?	☐ Yes, currently<br>☐ Yes, in the past, but I quit at least 6 months ago<br>☐ No, never
Do you use snuff?	☐ Yes ☐ No
What is your height (cm)?<br>What is your weight (kg)?	………<br>……….
Have you had any of following diseases diagnosed by a doctor (before you fell ill with COVID-19)? Tick one or more options.	☐ Hypertension<br>☐ Other heart disease<br>☐ Diabetes mellitus (type 1 or 2)<br>☐ Lung disease<br>☐ Liver disease<br>☐ Stroke<br>☐ Anxiety<br>☐ Depression<br>☐ Chronic pain (in the last year, symptoms for at least 3 months)<br>☐ Cancer with/without treatment<br>☐ Other disease with immunosuppressive treatment<br>☐ Hypo-/hyperthyroidism<br>☐ No
How would you rate your physical fitness relative to that among other people of the same age?	☐ Better<br>☐ Same<br>☐ Worse
How would you rate your physical fitness before COVID-19 relative to that among other people of the same age?	☐ Better<br>☐ Same<br>☐ Worse
Did you have any of these symptoms at onset? Tick one or more options.	☐ Fever<br>☐ Breathing problems<br>☐ Muscle/joint pain<br>☐ Sore throat<br>☐ Headache<br>☐ Impaired sense of smell and taste<br>☐ Nasal congestion

	☐ Cough<br>☐ Eye irritation<br>☐ Skin rash<br>☐ Pain in the chest<br>☐ Heart palpitation<br>☐ Anxiety<br>☐ Depression<br>☐ Gastrointestinal symptoms (nausea, diarrhea, stomach pain)<br>☐ Fatigue<br>☐ Dizziness<br>☐ No symptoms
Did you have any of these symptoms one month after your COVID-19 diagnosis? Tick one or more options.	☐ Fever<br>☐ Breathing problems<br>☐ Muscle/joint pain<br>☐ Sore throat<br>☐ Headache<br>☐ Impaired sense of smell and taste<br>☐ Nasal congestion<br>☐ Cough<br>☐ Eye irritation<br>☐ Skin rash<br>☐ Pain in the chest<br>☐ Heart palpitation<br>☐ Anxiety<br>☐ Depressed mood<br>☐ Gastrointestinal symptoms (nausea, diarrhea, stomach pain)<br>☐ Fatigue<br>☐ Dizziness<br>☐ No symptoms
Did you have any of these symptoms three months after COVID-19 diagnosis? Tick one or your more options.	☐ Fever<br>☐ Breathing problems<br>☐ Muscle/joint pain<br>☐ Sore throat<br>☐ Headache<br>☐ Impaired sense of smell and taste<br>☐ Nasal congestion<br>☐ Cough<br>☐ Eye irritation<br>☐ Skin rash<br>☐ Pain in the chest<br>☐ Heart palpitation<br>☐ Anxiety<br>☐ Depressed mood<br>☐ Gastrointestinal symptoms (nausea, diarrhea, stomach pain)<br>☐ Fatigue<br>☐ Dizziness<br>☐ No symptoms

Did you have any of these symptoms six months after your COVID-19 diagnosis? Tick one or more options.	☐ Fever<br>☐ Breathing problems<br>☐ Muscle/joint pain<br>☐ Sore throat<br>☐ Headache<br>☐ Impaired sense of smell and taste<br>☐ Nasal congestion<br>☐ Cough<br>☐ Eye irritation<br>☐ Skin rash<br>☐ Pain in the chest<br>☐ Heart palpitation<br>☐ Anxiety<br>☐ Depressed mood<br>☐ Gastrointestinal symptoms (nausea, diarrhea, stomach pain)<br>☐ Fatigue<br>☐ Dizziness<br>☐ No symptoms
Do you currently, twelve months after your COVID-19 diagnosis, have any of the symptoms that started in connection with the acute infection? Tick one or more options.	☐ Fever<br>☐ Breathing problems<br>☐ Muscle/joint pain<br>☐ Sore throat<br>☐ Headache<br>☐ Impaired sense of smell and taste<br>☐ Nasal congestion<br>☐ Cough<br>☐ Eye irritation<br>☐ Skin rash<br>☐ Pain in the chest<br>☐ Heart palpitation<br>☐ Anxiety<br>☐ Depressed mood<br>☐ Gastrointestinal symptoms (nausea, diarrhea, stomach pain)<br>☐ Fatigue<br>☐ Dizziness<br>☐ No symptoms
How do you experience your general health now? • *100 means that you are in the best possible health, 0 means that you are in the worst possible health.* • Tick one option.	☐ 100 ☐ 95 ☐ 90 ☐ 85 ☐ 80 ☐ 75<br>☐ 70 ☐ 65 ☐ 60 ☐ 55 ☐ 50 ☐ 45<br>☐ 40 ☐ 35 ☐ 30 ☐ 25 ☐ 20 ☐ 15<br>☐ 10 ☐ 5 ☐ 0
How did you experience your general health before COVID-19? • *100 means that you were in the best possible health, 0 means you were in the worst possible health.* • Tick one option.	☐ 100 ☐ 95 ☐ 90 ☐ 85 ☐ 80 ☐ 75<br>☐ 70 ☐ 65 ☐ 60 ☐ 55 ☐ 50 ☐ 45<br>☐ 40 ☐ 35 ☐ 30 ☐ 25 ☐ 20 ☐ 15<br>☐ 10 ☐ 5 ☐ 0

What do you do (primary occupation)? Tick one option.	☐ Working ☐ Parental leave ☐ Sick leave ☐ Unemployed/looking for a job ☐ Retired ☐ Student
If you are working, what is your primary occupation?	……
Have you sought healthcare (including phone call to a healthcare advisor, visit to primary care or secondary care) due to persistent symptoms after COVID-19?	☐ Yes ☐ No
A) Have you been on sick leave (with a doctor's certificate) due to COVID-19 or its consequences?<br>B) If "Yes," for how many weeks?	☐ Yes ☐ No<br>……………..
A) Were you on sick leave (with a doctor's certificate) before the pandemic (during 2019)?<br>B) If "Yes," for how many weeks?	☐ Yes ☐ No<br>……….
How would you rate your work ability at the moment, one year after your COVID-19 diagnosis? Please tick one option. Zero is the worst and ten is the best work ability.	☐ 0 ☐ 1 ☐ 2 ☐ 3 ☐ 4 ☐ 5 ☐ 6 ☐ 7 ☐ 8 ☐ 9 ☐ 10
How would you rate your work ability before the COVID-19 infection? Please tick one option. Zero is the worst and ten is the best work ability.	☐ 0 ☐ 1 ☐ 2 ☐ 3 ☐ 4 ☐ 5 ☐ 6 ☐ 7 ☐ 8 ☐ 9 ☐ 10
