# Supplementary material for: Predictors of post-COVID-19 and the impact of persistent symptoms in non-hospitalized patients 12 months after COVID-19, with a focus on work ability
Source: Ups J Med Sci. 2022 Aug 9;127:10.48101/ujms.v127.8794. doi: 10.48101/ujms.v127.8794 (PMC9383047; doi:10.48101/ujms.v127.8794)

Supplementary Figure 1. The distribution of symptoms (%) over time, from symptom onset through 1, 3, 6, and 12 months after a COVID-19 diagnosis in the working study population divided into those working in healthcare with patient contact and other occupational groups.

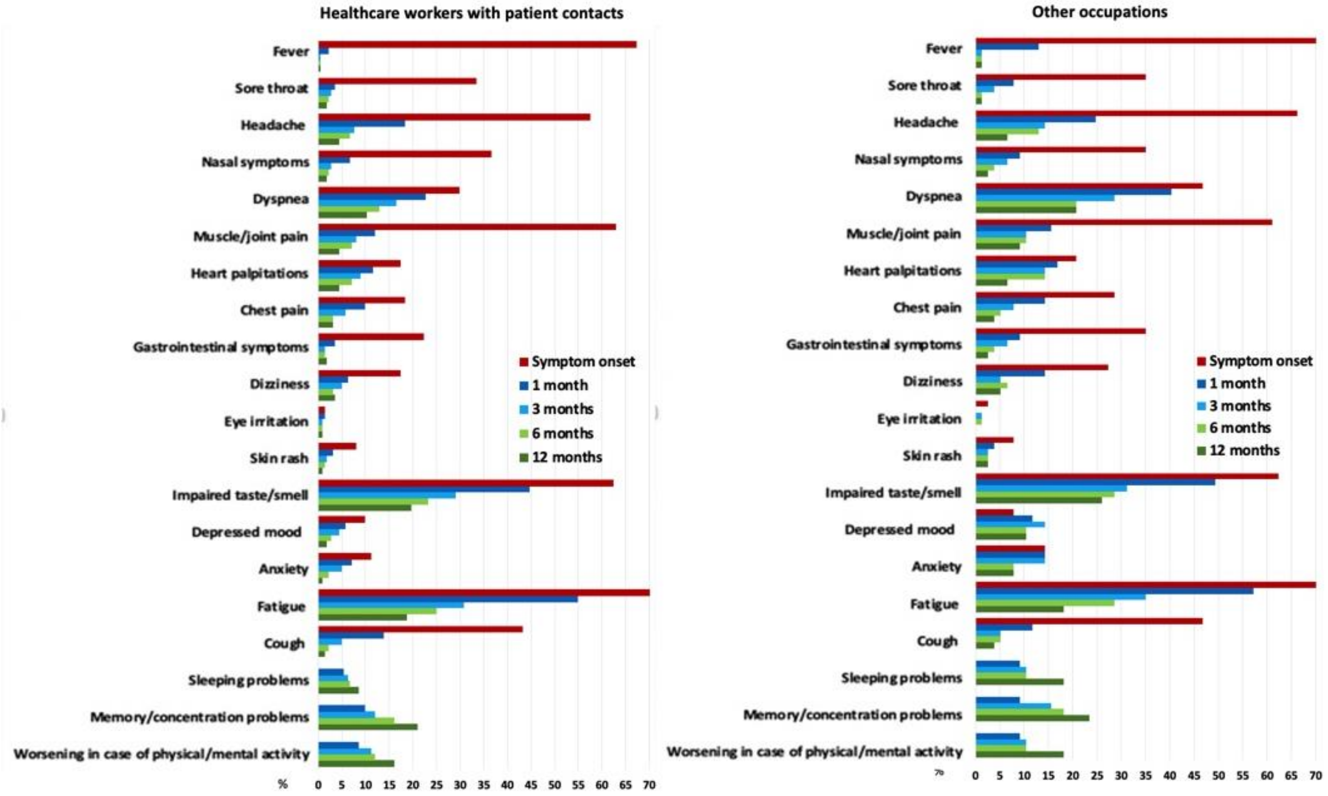

Supplement: Predictors of post-COVID-19 and the impact of persistent symptoms in non-hospitalized patients 12 months after COVID-19, with a focus on work ability [file UJMS-127-8794-s002.pdf]
