# Supplementary material for: Predictors of post-COVID-19 and the impact of persistent symptoms in non-hospitalized patients 12 months after COVID-19, with a focus on work ability
Source: Ups J Med Sci. 2022 Aug 9;127:10.48101/ujms.v127.8794. doi: 10.48101/ujms.v127.8794 (PMC9383047; doi:10.48101/ujms.v127.8794)
Supplement: Predictors of post-COVID-19 and the impact of persistent symptoms in non-hospitalized patients 12 months after COVID-19, with a focus on work ability [file UJMS-127-8794-s003.pdf]

Supplementary Table 1. Comorbidity of the study population divided into the occupational groups and groups with and without persistent symptoms at twelve months. Data presented as n and percent (%).

	**Healthcare with patient contact**		**Other occupational groups**	
Symptoms at twelve months	No<br>N=125	Yes<br>N=99	No<br>N=38	Yes<br>N=39
Hypertension	11 (9)	14 (11)	4 (10)	5 (13)
Other heart disease	2 (2)	1 (1)	1 (3)	2 (5)
Hypo-/hyperthyroidism	9 (7)	8 (6)	1 (3)	2 (5)
Diabetes mellitus	2 (2)	3 (2)	1 (3)	1 (3)
Lung disease	9 (7)	10 (8)	4 (10)	5 (13)
Immunosuppressive treatment	4 (3)	5 (3)	1 (3)	2 (5)
Cancer	3 (3)	4 (3)	2 (5)	2 (5)
Depression	15 (15)	16 (13)	6 (15)	4 (11)
Anxiety	12 (12)	11 (9)	10 (26)	6 (16)
Chronic pain	2 (2)	5 (3)	1 (3)	2 (5)
